# Electrochemical Approach to Measure Physiological Fluid Flow Rates

**DOI:** 10.3389/fchem.2021.680099

**Published:** 2021-06-16

**Authors:** Srivats Sarathy, Marco A. Nino, Abdulsattar H. Ghanim, Srinivasan Rajagopal, Syed Mubeen, M. L. Raghavan

**Affiliations:** ^1^ Roy J Carver Department of Biomedical Engineering, University of Iowa, Iowa City, IA, United States; ^2^ Department of Chemical and Biochemical Engineering, University of Iowa, Iowa City, IA, United States; ^3^ Department of Anesthesia, University of Iowa Hospitals and Clinics, Iowa City, IA, United States

**Keywords:** flow measurement, electrochemistry, blood flow, ac voltammetry, electrodilution

## Abstract

*In vivo* measurement of the flow rate of physiological fluids such as the blood flow rate in the heart is vital in critically ill patients and for those undergoing surgical procedures. The reliability of these measurements is therefore quite crucial. However, current methods in practice for measuring flow rates of physiological fluids suffer from poor repeatability and reliability. Here, we assessed the feasibility of a flow rate measurement method that leverages time transient electrochemical behavior of a tracer that is injected directly into a medium (the electrochemical signal caused due to the tracer injectate will be diluted by the continued flow of the medium and the time response of the current—the electrodilution curve—will depend on the flow rate of the medium). In an experimental flow loop apparatus equipped with an electrochemical cell, we used the AC voltammetry technique and tested the feasibility of electrodilution-based measurement of the flow rate using two mediums—pure water and anticoagulated blood—with 0.9 wt% saline as the injectate. The electrodilution curve was quantified using three metrics—change in current amplitude, total time, and change in the total charge for a range of AC voltammetry settings (peak voltages and frequencies). All three metrics showed an inverse relationship with the flow rate of water and blood, with the strongest negative correlation obtained for change in current amplitude. The findings are a proof of concept for the electrodilution method of the flow rate measurement and offer the potential for physiological fluid flow rate measurement *in vivo*.

## Introduction

“It was fatal for the development of our understanding of circulation that blood flow is relatively difficult to measure while blood pressure so easy to measure … although most organs do not need blood pressure, but flow” (Dünser et al., 2013). In 1928, physician-scientist Jarisch aptly summarized this problem in medicine that has endured to this day. And it is not just the measurement of the rate of blood flow, but that of most physiological flows—the cerebrospinal fluid flow is another such example. Almost a 100 years after Jarisch, it is fair to say, the difficulty in measuring physiological flow with accuracy and precision continues to elude us (Pironet et al., 2016; McKenzie et al., 2018). Consider, for instance, the cardiac output (CO)–the blood flow rate in the heart. CO serves as a key aggregate marker of cardiac function during many surgical procedures and for estimating cardiopulmonary function in a hospital’s cardiac catheterization laboratory or the intensive care unit. Yet, unlike synthetic fluids flowing through pipes, the human body presents limited access, flow through arbitrarily shaped structures, material heterogeneity, and significant risks. These challenges impede our ability to model, calibrate, and measure fluid flow rates *in vivo*.

Over the years, several methods have been developed to measure physiological flow rates. Some of these include methods that use an indicator dilution principle, such as thermodilution or dilution of labeled particles/microspheres (Heymann et al., 1977; Marcus et al., 1987; Mehta, 2014), and others that are noninvasive, such as the use of electromagnetic probes, bioimpedance, or ultrasonic Doppler methods (Franklin, 1965; Mehta, 2014). Thermodilution is the most common technique used in the clinic for blood flow measurement in the heart—the cardiac output. A pulmonary artery catheter is advanced through the right atrium and placed in the pulmonary trunk. A bolus of cooled saline (the injectate) is injected into the right atrium through a proximal port. The saline mixes with blood and causes a slight drop (usually less than 1°F) in temperature. As the cooled blood–injectate mix passes through the pulmonary trunk, the temperature drop is sensed from a thermistor on the catheter tip. As more blood flows, the temperature will rise back up to preinjection levels. This temporal fall and subsequent rise in temperature—thermodilution—will depend on blood flow rate, which may be recovered through calibration (Callister and Machold, 2006). There is, however, poor reproducibility with this technique because the temperature can be affected by numerous confounding factors such as changes to saline temperature during injection, breathing, etc. Less invasive measurement techniques such as Doppler ultrasound are highly operator sensitive with even poorer reliability (Franklin, 1965; Mehta, 2014). In a 2016 report, Pironet et al. (2016) compared the four most common techniques used (including thermodilution) under controlled settings. They concluded that “*cardiac output estimate provided by any technique relates poorly to the estimates from the others*.” Thermodilution is thought to have a precision error of 20% (Stetz et al., 1982), and with the gold standard itself with such poor precision, its alternatives are evaluated for acceptability, when they have less than 30% error (Critchley and Critchley, 1999). There is, therefore, a need for a more reliable *in vivo* measurement of physiological fluid flow rate.

**FIGURE 1:**
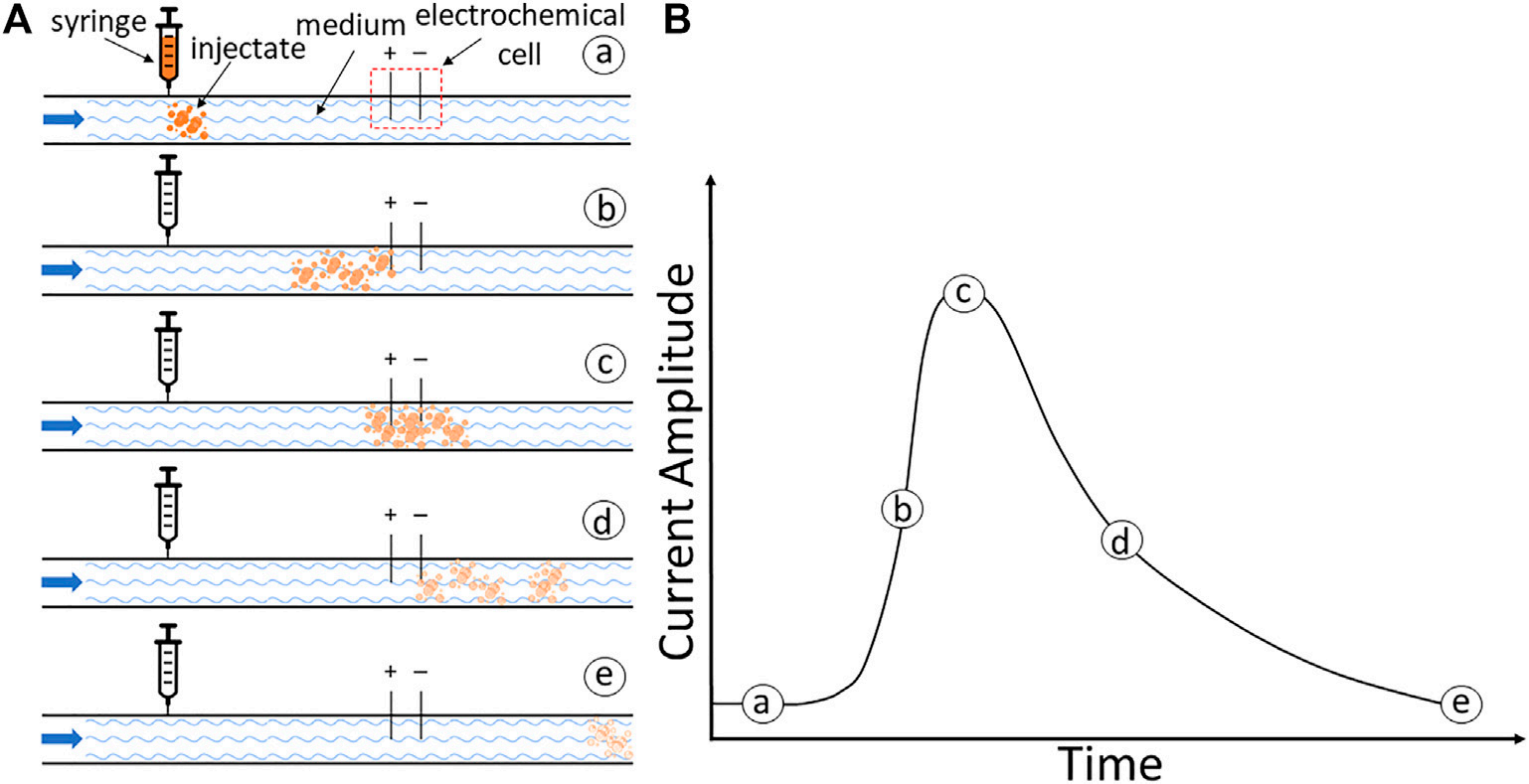
**(A)** Schematic illustration of the electrodilution principle. **(A)** Injection of tracer. **(B)** Initiation of the current response as the mixture reaches the electrochemical cell. **(C)** Peak response to the injectate mixture. **(D)** Dilution by the continued flow of the medium. **(E)** Return to the baseline. **(B)** A model current time response with currents.

Here, we developed an alternative approach—the electrodilution method—that is operationally similar to thermodilution but leverages electrochemical principles. In electrodilution, when a small voltage is applied to the flowing media, the introduction and dilution of an injectate will result in a charge transfer (faradaic process) and/or charge redistribution (capacitive process) and release a measurable current change (Park et al., 2019). These temporal changes in current—current dilution—will be related to the blood flow rate because the greater the flow, the quicker the dilution. The flow rate of the media could then be recovered by appropriate calibration. This is schematically illustrated in Figure 1. (a) The tracer is injected which mixes with the flowing medium, following which (b) the mixture reaches the electrochemical cell and causes a change in the current response. (c) The temporary change in the electrical current causes a peak current. (d) The flowing medium will dilute this response, and (e) the measured current eventually returns to the baseline. The strength of the response and its dilution time will be affected by the volumetric flow rate of the flowing fluid medium. Thus, the above dilution curve can be used to recover the medium’s flow rate. This study aimed to assess proof of concept for the electrodilution method and develop metrics that best permit the recovery of the blood flow rate.

## Materials and Methods

### Materials

Blood samples were obtained as needed from Lampire Biological Products (Pipersville, PA United States) and a local abattoir. Chemicals were purchased from Research Products International (Mount Prospect, IL United States): sodium citrate (Na_3_C_6_H_5_O_7_, 99%). Sodium chloride solution (NaCl, 0.9 wt%) was obtained from Baxter Healthcare Corporation (Deerfield, IL United States). Deionized water was used as the baseline media for all experiments.

### Experimental Setup

**FIGURE 2:**
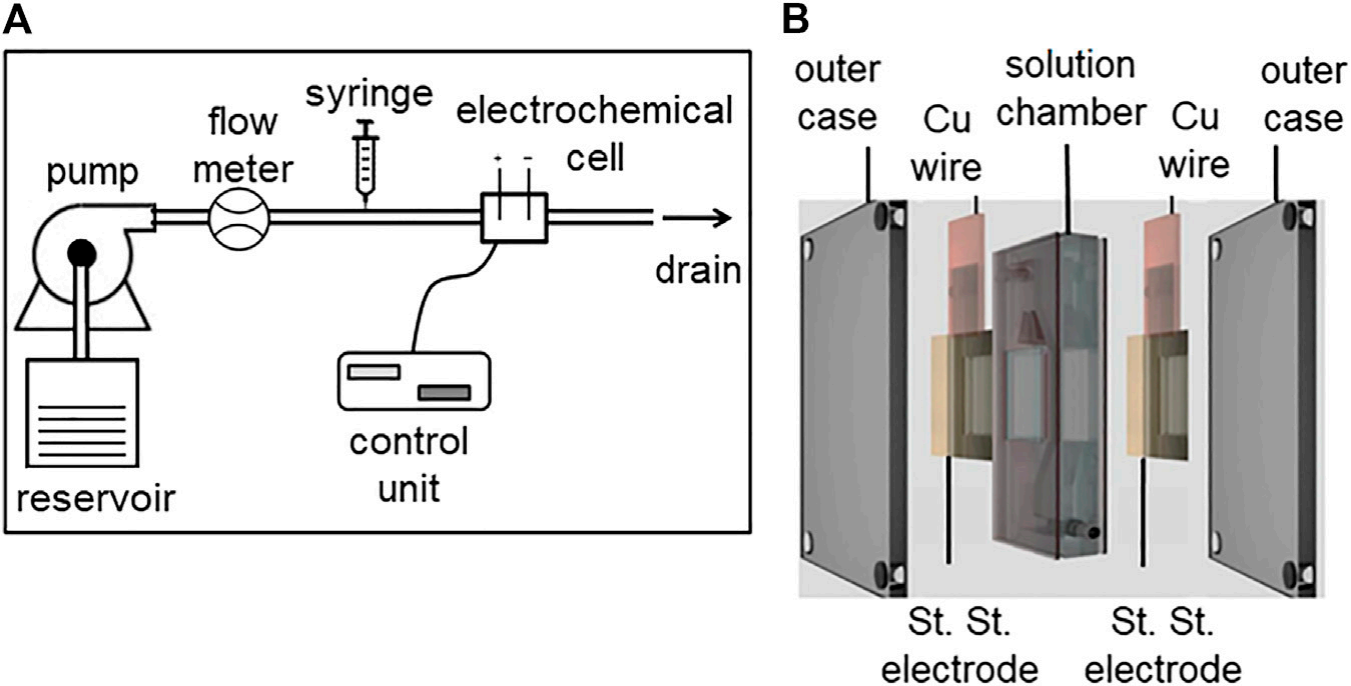
**(A)** Schematic of the experimental flow loop apparatus and **(B)** electrochemical cell.

An experimental apparatus with a predetermined flow of a medium through an electrochemical cell, with the ability to inject a precise amount of indicator is shown in Figure 2A. A continuous flow pump (75211–62 variable speed pump drive, Cole-Parmer Instrument Company LLC., Burlington, IL United States) along with an inline flow meter (Masterflex direct reading variable area flow meter, Cole-Parmer Instrument Company LLC., Vernon Hills, IL United States) was used to sustain the predetermined flow rate. A syringe pump (KDS-210, KD Scientific Inc., Houston, MA United States) connected in series with the flow loop was used to control the release of injectate. The inline electrochemical cell (Figure 2B) used a two-electrode configuration, with stainless steel as the working and counter electrode of area 2.25 cm^2^. Stainless steel was chosen as the preferred electrode material because of its biocompatibility in blood and high chemical/electrochemical corrosion resistance (Hedberg and Wallinder, 2016). The electrodes were connected to a potentiostat control unit (VSP-300, BioLogic Science Instruments, Seyssinet-Pariset, France). The two electrodes were separated by a solution chamber (7 ml) with an inlet and outlet. For all measurements, a sinusoidal voltammetry technique was used to measure electrochemical perturbations caused by the injectate.

### Electrodilution Experiments

Experiments were conducted at different flow rates to document how the electrodilution curves are affected by the flow rate of the medium. Two fluid mediums—pure water and anticoagulated bovine blood—were studied. Saline (0.9 wt% NaCl) was selected as the injectate as it is clinically used in thermodilution. The electrochemical cell was connected to a potentiostat control unit. A sinusoidal potential waveform (0.1 V_pk_ and 0.5 kHz) was applied to the electrochemical cell, and the corresponding current signal was continuously recorded. The pump was set to a predetermined flow rate, and the medium was allowed to flow until the measured current across the electrodes reached equilibrium. This nominal value for current serves as the baseline. Next, a syringe pump delivered 1.5 ml of injectate over 6 s into the flowing medium proximal to the electrochemical cell. The volume and flow rate of injectate is lower than that used during thermodilution-based operations, and therefore, is known to be safe for clinical use (Callister and Machold, 2006; Pironet et al., 2016). The resulting current response and recovery were recorded. After a short delay to allow for the current recovery, the injection was repeated at that same flow rate. The process was repeated for various medium flow rates ranging from 100 to 600 ml/min, which constitute the typical blood flow rates in pediatric subjects (Emmanouilides et al., 1970). To further assess how the sinusoidal potential waveform parameters impact electrodilution measurements, additional amplitude and frequency combinations (0.25 kHz and 0.01 V_pk_, 0.5 kHz and 0.01 V_pk_, and 0.5 kHz and 0.1 V_pk_) were also studied.

Experiments were also conducted using anticoagulated bovine blood (9:1 bovine blood to 3.2 wt% Na-citrate) as the fluid medium. In contrast to the water medium experiments, the blood medium experiments required a closed loop design to save blood volume and a peristaltic pump (YZ151x, Longer Precision Pump Co., Ltd., Hebei, China) for noncontact pumping. The outlet from the electrochemical cell was connected to the inlet of the pump—completing the closed loop design. Electrodilution experiments with saline injectate were conducted with blood as the medium for predetermined flow rates of 100, 200, and 300 ml/min. The same sinusoidal potential waveform with amplitude and frequency parameters of 0.1 V_pk_ and 0.5 kHz, respectively, were used.

## Results and Discussion

The intended application of this method is to recover the flow rate of blood through saline injections. However, experiments with blood *ex vivo* are complex and cumbersome owing to their tendency to clot. Therefore, we first conducted experiments with saline injections in water as the flowing media to assess the electrodilution proof of concept and identify appropriate AC voltammetry settings (voltage amplitude and frequency). Subsequently, controlled experiments were performed at chosen voltammetry settings with saline injections in animal blood as flowing media to corroborate our findings under realistic conditions.

### Electrodilution Experiments With Water as the Flowing Media

**FIGURE 3:**
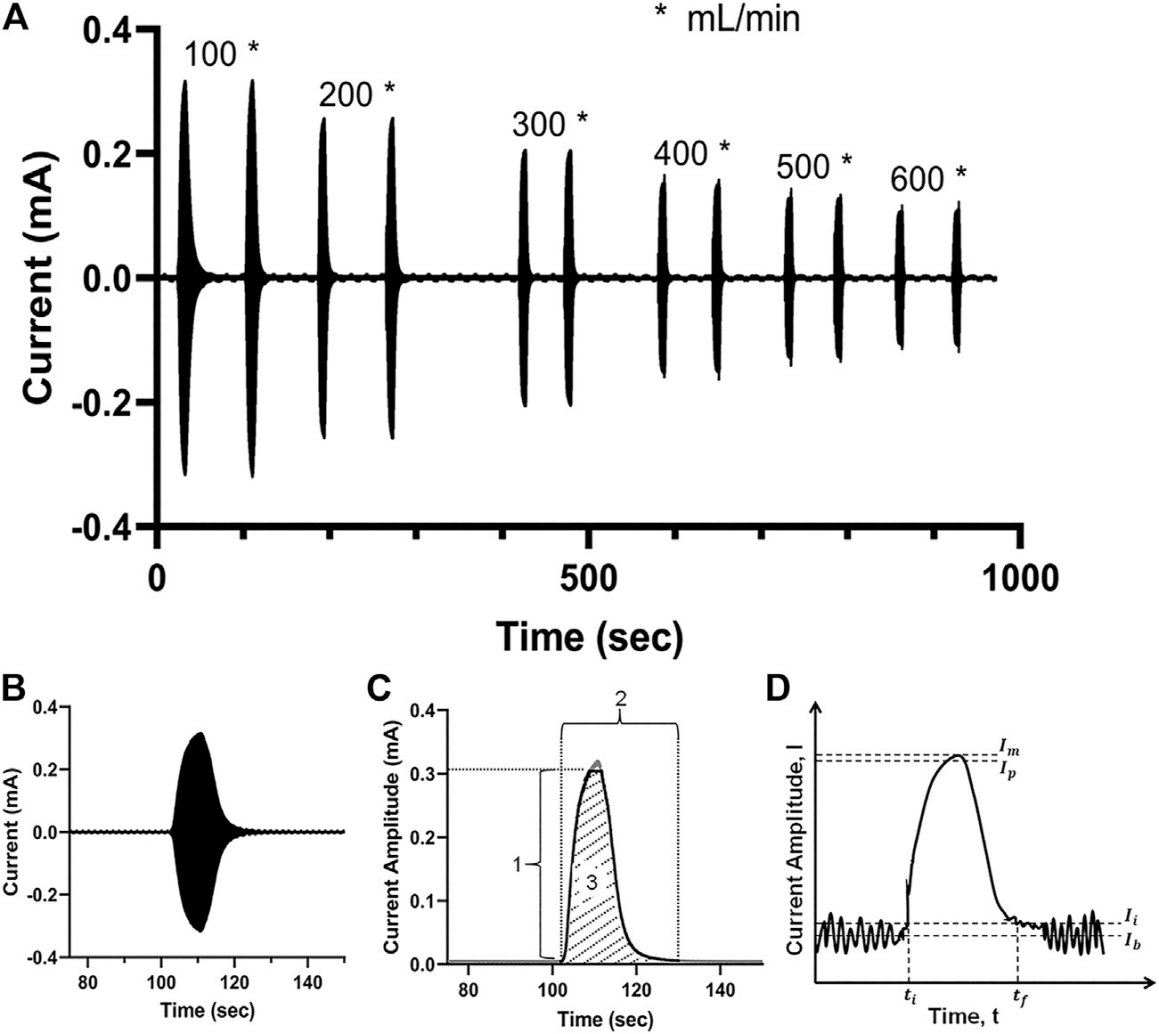
**(A)** Measured current signal over time for a pair of saline injections at each of six flow rates for water—from 100 to 600 ml/min. **(B)** The measured current signal after a single injection; the densely packed sinusoidal AC signal appears as a filled curve; **(C)** the crest of the current signal amplitude is extracted to obtain the electrodilution curve (solid black line); 1: Δ current amplitude; 2: total time; 3: Δ total charge. **(D)** The electrodilution metrics are determined from the electrodilution curve shown (baseline noise exaggerated for illustration purposes). All measurements were carried out at AC voltammetry settings of 0.1 V_pk_ and 0.5 kHz.

Figure 3A illustrates a proof-of-concept electrodilution experiment using water as the flowing media and 0.9 wt% saline as the injectate. Six different flow rates (100–600 ml/min) were studied with two injections of saline for each flow rate. A sinusoidal voltammetry technique—where the working electrode is excited using a potential sinusoidal waveform—was applied to measure the current response (see “Methods” for more details). The AC voltammetry technique was chosen to capture double-layer charging/discharging currents due to the adsorption of injectate tracer molecule near the sensing electrode’s surface, with high sensitivity. We found that for all flow rates, the release of saline into the flowing medium not only resulted in a detectable signal but also had a typical dilution curve—the steep rise and gradual fall back to the baseline (Figure 3B). These results are consistent with the proposed principle of electrodilution.

The temporal current data were post-processed using a semiautomated algorithm to identify the metrics that best correlate the changes in electrochemical signal to flow rates. For post-processing, only the positive portion of the current response was utilized. The electrodilution curve is represented by the crests of the current signal, which constitutes both the response (rise) and recovery (fall) phases of the electrochemical perturbation introduced by the injectate (see shaded region 3 in Figure 3C). To quantitatively assess the relationship between the medium flow rate and the electrodilution curve, three metrics were calculated from the curve: 1) Δ *current amplitude* is the change in current amplitude (see Eq. 1) which is defined as 95% of the maximum change from initial current (*Ii*). *Ii* is defined as 2.5 standard deviations above average baseline current (as shown in Figure 3D). The 95% of the maximum change was selected to minimize the effect from current spike artifacts at its peak. 2) *Total time* is defined as the total time taken for the measured current to rise from the baseline and fall back to the baseline (see Eq. 2). 3) Δ *total charge* is defined as the charge observed during the duration of the rise from the baseline and fall back to baseline of current (area under the dilution curve (see Eq. 3)). More quantitative definitions follow and are illustrated in Figure 3D.

$$\Delta \;current\;amplitude\;=\;I_{p}-I_{i},$$ (1)

$$Total\;time\;=\;t_{f}-t_{i},$$ (2)

$$\Delta \;total\;charge\;=\int_{t_{i}}^{t_{f}}\left(I-I_{i}\right)\;.$$ (3)

Here, $I_{b}$ : average of baseline current before and after the dilution curve
$I_{i}$ : $I_{b}+2.5\cdot SD$ of baseline current amplitude before and after the dilution curve
$I_{m}$ : maximum current reached in the dilution curve
$I_{p}$ : peak current = $0.95\left(I_{m}-I_{i}\right)+I_{i}$
$t_{i}$ : start time of the dilution curve when current breaches $I_{i}$
$t_{f}$ : end time of the dilution curve when current falls below $I_{i}$

Note: Δ total charge is calculated by truncating the dilution curve at $I_{p}$ to minimize effect from any current spike artifact.

**FIGURE 4:**
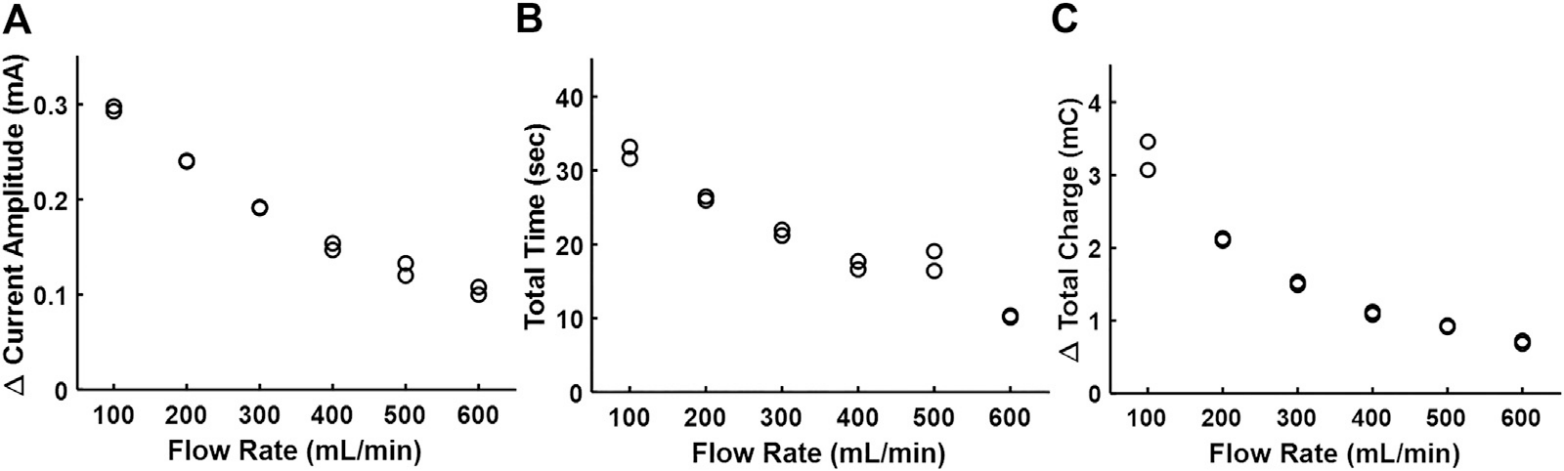
Effect of the increasing flow rate of water on the electrodilution metrics. **(A)** Δ Current amplitude, **(B)** total time, and **(C)** Δ total charge. Two saline injections were administered for each flow rate (note two data points per flow rate). AC voltammetry settings were 0.1 V_pk_ and 0.5 kHz.

Figure 4 shows the correlation of the three metrics of electrodilution—change in current amplitude, total time, and total charge—as a function of water flow rates. All three metrics showed an inverse relationship with the flow rate for a given sinusoidal voltage amplitude and frequency (0.1V_pk_ and 0.5 kHz). The finding that Δ current amplitude reduces with a greater volumetric flow rate could be explained by considering the interactions of fluid dynamics and electrochemistry. For a constant injection flow rate and volume, the concentration of the injectate in the medium that reaches the electrode surface will decrease with an increase in the medium flow rate (Cheng, 2009). The electrochemical importance of the surface concentration of the injectate (here, concentration of Na^+^ and Cl^−^ in the injectate saline) is directly related to the current. Hence, higher flow rates will result in lower electrochemical currents (Figure 4A). In addition, a greater flow rate of the medium will also accelerate its dilution and consequently result in a lower total dilution time (Figure 4B) and lower charge (Figure 4C; area under the electrodilution curve). These results are consistent with the premise that the medium flow rates can be recovered from one or all of the three metrics of the electrodilution curve.

Table 1 summarizes the dependence of the three electrodilution metrics for different potential waveform amplitude and frequency combinations with water as the flowing medium. The reported value at every flow rate is the average of the two values measured during the paired injections. Across all the experiments, the measured metrics follow a decreasing trend with an increasing flow rate. With an increase in frequency and potential amplitude, an increase in sensitivity to the measured current signal was observed, consequently improving the correlation between the three metrics and medium flow rates. Based on these results, a potential waveform amplitude and frequency of 0.1 V_pk_ and 0.5 kHz was chosen for blood experiments as it gave the most favorable negative correlation for Δ current amplitude with the flow rate.

**TABLE 1:** Effect of AC voltammetry settings on electrodilution metrics.

	Δ Current amplitude (mA)			Total time (sec)			Δ Total charge (mC)		
Flow rate (ml/min)	0.01 V_pk_ 0.25 kHz	0.01 V_pk_ 0.5 kHz	0.1 V_pk_ 0.5 kHz	0.01 V_pk_ 0.25 kHz	0.01 V_pk_ 0.5 kHz	0.1 V_pk_ 0.5 kHz	0.01 V_pk_ 0.25 kHz	0.01 V_pk_ 0.5 kHz	0.1 V_pk_ 0.5 kHz
100	0.0071	0.0068	0.2955	36.97	18.50	32.42	0.1185	0.0645	3.265
200	0.0068	0.0061	0.2405	23.96	16.90	26.20	0.0730	0.0510	2.115
300	0.0062	0.0051	0.1915	17.36	16.05	21.57	0.0590	0.0455	1.510
400	0.0051	0.0047	0.1505	16.89	14.27	17.16	0.0485	0.0370	1.100
500	0.0048	N/A	0.1265	11.82	N/A	17.75	0.0395	N/A	0.920
600	0.0043	N/A	0.1040	11.88	N/A	10.24	0.0352	N/A	0.700
Correl	−0.975	−0.983	−0.983	−0.897	−0.588	−0.968	−0.917	−0.980	−0.937

**FIGURE 5:**
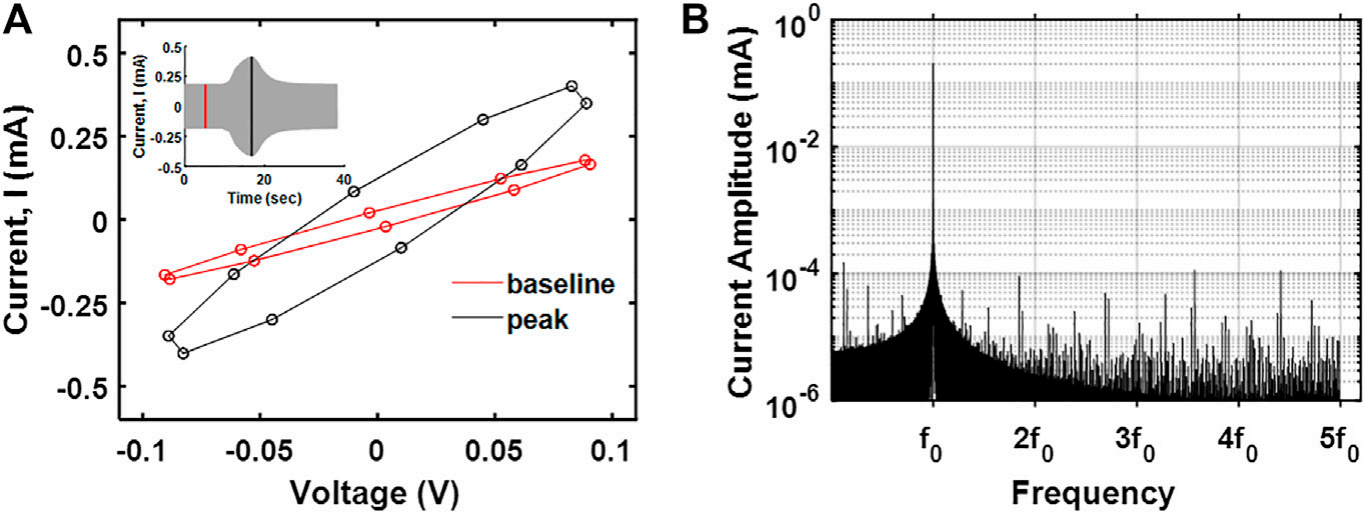
**(A)** *I*–*V* curve at the peak response and baseline for saline electrodilution in water. **(B)** The frequency spectrum of the transient current measured showing the first five harmonics.

We then investigated the relative contribution of faradaic (e.g., electron transfer) and non-faradaic (e.g., capacitive) processes on the nature of currents generated due to saline injections. The data acquisition rate was ten times the fundamental frequency (0.5 kHz) to facilitate the identification of higher order harmonics in the data. For a qualitative assessment, current-potential data were visualized (Figure 5A). The current potential data obtained after saline injection were symmetrically disposed, indicating the currents generated were predominantly capacitive. For a quantitative assessment, the frequency domain for the current signal was analyzed to separate faradaic and non-faradaic contributions [the faradaic currents are better represented at fifth or higher order harmonics, and lower order of harmonics is associated with capacitive currents (Brazill et al., 2002)]. As shown in Figure 5B, the peak currents at fifth-order harmonics are negligible to that at the fundamental frequency, suggesting that the currents observed upon saline injection are primarily due to capacitive contributions. This has some implications for the recovery of flow rates of physiological fluids. While faradaic processes could enable us to capture electrochemical signatures specific to the injectate (desirable), it holds the risk of altering the chemistry of the physiological fluid (nondesirable).

### Electrodilution Experiments With Blood as the Flowing Media

**FIGURE 6:**
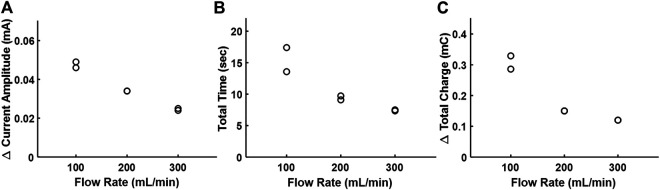
Effect of the increasing flow rate of bovine blood on the electrodilution metrics. **(A)** Δ Current amplitude, **(B)** total time, and **(C)** Δ total charge. All measurements were carried out at AC voltammetry settings of 0.1 V_pk_ and 0.5 kHz.

Figure 6A shows the effect of the bovine blood flow rate on the electrodilution metrics at 0.1 V_pk_ and 0.5 kHz as chosen from the water medium experiments. As with water, Δ current amplitude, total time, and total charge showed an inverse correlation with increased blood flow rate. Among the three metrics, the Δ current amplitude showed the strongest negative correlation (−0.990) with the flow rate. However, the absolute value of Δ current amplitude in blood was an order of magnitude lower than that observed with water due to the increased ionic conductivity of blood, resulting in increased baseline currents. These results indicate that the saline electrodilution method can be used to recover blood flow rates.

The principle of using electrochemical properties of blood with select electrolyte solutions to determine the blood flow rate *in vivo* has been studied (Stewart, 1897; White, 1947; Geddes, 1974, 1989; Bourdillon et al., 1979; Grubbs et al., 1984). However, these prior reports were based on measuring the changes in electrolytic conductivity of blood, which has not been adopted due to its limited reliability attributed to modest signal-to-noise ratio and calibration difficulties (White, 1947; Bourdillon et al., 1979; Ehlers et al., 1986). In this study, we utilize advancements in AC voltammetry techniques (use of the large amplitude sinusoidal voltammetry technique) that allowed us to investigate different characteristics of an induced electrical current change and use a combination of metrics to model/optimize estimations of the volumetric flow rate using electrodilution with significantly greater reliability, thereby enabling us to sidestep challenges associated with the temperature measurement. Furthermore, the LASV technique allowed us to decouple the current contributions from faradaic and non-faradaic processes, potentially opening up opportunities to measure flow rates of redox-active biological species selectively. If electrodes optimally designed for electrodilution may be fabricated onto catheters (this is quite feasible as they already are in thermodilution catheters), the ability to measure flow rates of physiological fluids such as blood flow in the heart or cerebrospinal fluids with reliability is feasible.

## Data Availability

The original contributions presented in the study are included in the article/Supplementary Material; further inquiries can be directed to the corresponding authors.
