# Supplementary figures and images for: Electrochemical Approach to Measure Physiological Fluid Flow Rates
Source: Front Chem. 2021 Jun 16;9:680099. doi: 10.3389/fchem.2021.680099 (PMC8256275; doi:10.3389/fchem.2021.680099)

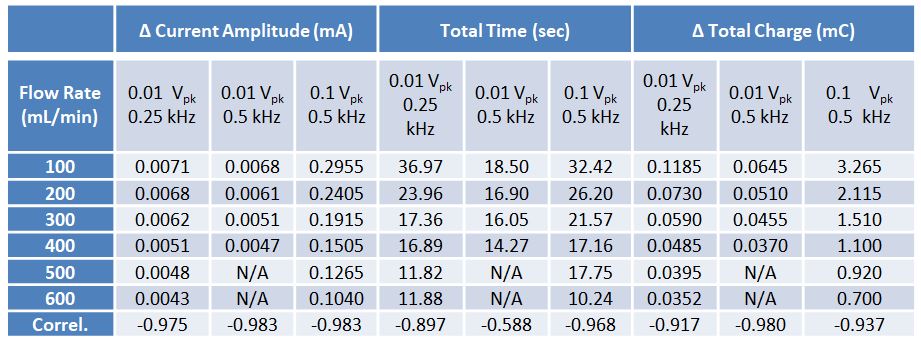

Supplement: Supplementary file 1 [file Image1.JPEG]
